# Urinary [TIMP-2]*[IGFBP7] for early prediction of acute kidney injury after coronary artery bypass surgery

**DOI:** 10.1186/s13613-015-0076-6

**Published:** 2015-12-15

**Authors:** Kevin Pilarczyk, Michaela Edayadiyil-Dudasova, Daniel Wendt, Ender Demircioglu, Jaroslav Benedik, Daniel Sebastian Dohle, Heinz Jakob, Fabian Dusse

**Affiliations:** Department of Thoracic and Cardiovascular Surgery, West German Heart and Vascular Center Essen, University Hospital Essen, Hufelandstr. 55, 45122 Essen, Germany

**Keywords:** Acute kidney injury, CABG, Coronary artery bypass surgery, Cardiac surgery, Biomarker, Insulin-like growth factor-binding protein 7, IGFBP7, Tissue inhibitor of metalloproteinases-2, TIMP-2

## Abstract

**Background:**

Acute kidney injury (AKI) is a common complication after cardiac surgery. Currently, prediction of AKI with classical tools remains uncertain. Therefore, it was the aim of the present study to evaluate two new urinary biomarkers—insulin-like growth factor-binding protein 7 (IGFBP7) and tissue inhibitor of metalloproteinases-2 (TIMP-2) in patients after coronary artery bypass surgery (CABG).

**Methods:**

In a prospective cohort study, 60 consecutive patients undergoing isolated on-pump CABG were enrolled. Urine samples collected every 12 h in the postoperative course were analyzed for the product of TIMP-2 and IGFBP7. Urinary output, serum creatinine and estimated glomerular filtration rate (eGFR) were recorded simultaneously. Primary clinical endpoint was the development of AKI stage 2 or 3 according to the classification of the KDIGO within 48 h after surgery.

**Results:**

48 male and 12 female patients with a mean age of 69.61 ± 8.4 years were included. 19 patients developed an AKI (31.6 %), six patients met the endpoint with AKI 2 or 3 (10 %). Urinary [TIMP-2]*[IGFBP7] increased significantly as early as 4 h after CABG in patients with AKI 2/3 (1.83 ± 2.15 vs. 0.23 ± 0.45, *p* < 0.05) whereas serum creatinine did not increase until 48 h after surgery. The diagnostic accuracy of [TIMP 2]*[IGFBP7] on day one after surgery for the prediction of AKI 2/3 was significantly better (sensitivity 0.89, specificity 0.81, AUC 0.817, 95 % CI 0.622–1.0 SE 0.099, *p* = 0.022, cut-off 0.817) than for serum creatinine (AUC 0.359, sensitivity 0.50, specificity of 0.52, cut-off value 1.17 mg/dl) and eGFR.

**Conclusion:**

Urinary [TIMP-2]*[IGFBP7] represents a sensitive and specific biomarker to predict moderate to severe AKI very early after CABG. Analyses from our ongoing larger study are necessary to confirm these findings and probably increase sensitivity and specificity.

## Background

Acute kidney injury (AKI) is a common and serious complication after coronary artery bypass grafting (CABG). According to current definitions, AKI occurs in up to 40 % of all patients undergoing cardiac surgery [1]. AKI requiring renal replacement therapy has an incidence of approximately 1–5 % [1]. The development of a postoperative AKI is associated with a more complicated clinical course and with an increased mortality of 15–30 %. Moreover, even slight changes of renal function are considered as an independent predictor of 30-day mortality [2]. Full recovery of renal function is only observed in 50 % of surviving patients [3, 4]. Thus, due to its significance, the term cardiac surgery-associated acute kidney injury (CSA AKI) was established. Risk assessment for AKI is recommended by clinical practice guidelines but remains imprecise mainly due to very limited sensitivity and specificity of early diagnostic tests available for AKI. Although serum creatinine is known to be an inadequate and delayed marker of acute changes in renal function, it is currently accepted as “gold standard” to diagnose AKI due to the lack of other reliable biomarkers. Obviously, there is a clear need for sensitive and specific biomarkers allowing early identification of patients with high risk for AKI to initiate preventive or therapeutic interventions [5–7].

Tissue inhibitor of metalloproteinases-2 (TIMP-2) and insulin-like growth factor binding protein 7 (IFGBP7) have recently been suggested as promising tools for the early detection of AKI in critically ill patients [8]. Both proteins are inducers of the G1 cell cycle arrest, considered as a key mechanism of AKI. In the Sapphire study enrolling 744 critically ill medical and surgical subjects with a moderate–severe AKI occurring in 14 % of patients, the combination of TIMP-2 and IGFBP7 was significantly superior to all other existing markers of AKI with an AUC of 0.80 and also better than TIMP–2 (AUC 0.76) and IGFBP7 (AUC 0.79) alone [9]. Others could confirm these findings showing that urinary [TIMP-2]*[IGFBP7] greater than 0.3 (ng/ml)^2^/1000 is able to identify patients at risk for imminent AKI [10]. In addition, urinary [TIMP-2]*[IGFBP7] is able to predict AKI and renal recovery early after cardiac surgery in a subgroup of patients with a high risk for the development of AKI defined by a high Cleveland Foundation Score [11]. Therefore, it was the aim of the present study to analyze the postoperative course of [TIMP 2]*[IGFBP7] to evaluate a new diagnostic approach for early detection of AKI in patients after isolated CABG.

## Methods

### Patients

The present study was approved by the Institutional Ethic Review Board and informed consent from the patient or the patient’s next of kin was obtained. Sixty consecutive patients (minimum age: 18 years) scheduled for elective on-pump CABG due to severe coronary artery disease between January 2014 and October 2014 were consecutively recruited during their pre-admission attendance to participate in the study. We used the Standards for Reporting of Diagnostic Accuracy (STARD) statement for planning and conducting the study and preparing the manuscript [12].

### Surgical procedure

Surgical revascularization was performed in all patients by using hypothermic cardiopulmonary bypass with cardioplegic arrest and median sternotomy as described previously [13]. Briefly, cannulation of the ascending aorta and two-stage venous cannulation was performed and standard nonpulsatile cardiopulmonary bypass (CPB) technique with a membrane oxygenator was initiated. During CPB, moderate hemodilution with a hematocrit between 20 and 25 % using mild systemic hypothermia (32 °C) was maintained. Myocardial protection was performed using anterograde cold crystalloid Bretschneider solution (Custodiol, Köhler Chemie GmbH, Bensheim, Germany) for cardioplegic arrest with additional topical cooling and single aortic crossclamping for all distal anastomoses.

### Biomarker measurements

Urine and blood samples for biomarker analysis were obtained 4 h after surgery and then every 12 h until discharge from ICU/IMC or for a maximum of 4 days, respectively. TIMP-2 and IGFBP7 in the urine were measured with the commercially available and FDA-approved NephroCheck™ Test (Astute Medical, San Diego, CA, USA)—a point-of-care test kit developed to measure and calculate the product of [TIMP-2] and [IGFBP7] concentration in the urine. Physicians in charge were blinded for the cell cycle arrest biomarker levels as well as laboratory investigators were blinded to clinical outcomes. Glomerular filtration rate was calculated with the Cockcroft–Gault formula (estimated Glomerular filtration rate = eGFR).

### Definition of endpoint and outcomes

AKI stage was determined daily on the basis of diuresis rate and serum creatinine concentration according to the Kidney Disease: Improving Global Outcomes classification (KDIGO) as follows [14]:

1. increase of serum creatinine by ≥0.3 mg/dl (≥26.4 µmol/L) or increase to ≥150–200 % from baseline or urine output <0.5 ml/kg/h for >6 h;
2. increase of serum creatinine to >200–300 % from baseline and/or urine output <0.5 ml/kg/h for >12 h;
3. increase of serum creatinine to >300 % from baseline or serum creatinine ≥4.0 mg/dl (≥354 µmol/L) after a rise of at least 44 µmol/L or treatment with renal replacement therapy and/or urine output <0.3 ml/kg/h for >24 h or anuria for 12 h.

The primary endpoint was the new occurrence of acute kidney injury stage 2 or 3 within 48 h after surgery. This observation period was chosen because prior studies have shown that AKI occurs within the first 24–72 h after CABG in the majority of cases.

Sepsis and septic shock was defined according to the definition issued by the Surviving Sepsis Campaign [15]. Shock was distinguished as cardiogenic shock (low cardiac output syndrome) or septic shock. Low cardiac output syndrome (LCOS) was defined as the need for an intra-aortic balloon pump (IABP) in the operating room (to be weaned from cardiopulmonary bypass) or in the intensive care unit because of hemodynamic compromise or the need for inotropic medication (epinephrine >0.25 µg/kg BW/min or milrinone >1.5 mg/h) to maintain systolic blood pressure greater than 90 mmHg and cardiac output index greater than 2.2 l/min/m^2^ for at least 30 min in the intensive care unit, after optimizing preload and afterload, correction of serum electrolyte, and blood gas abnormalities.

### Sample size calculation

The aim of the present study was to proof the hypothesis that mean urine [TIMP-2]*[IGFBP7] levels in patients with AKI differ significantly to patients without AKI within the first 24 h after surgery.

Therefore, a one-tailed power analysis based on the available published data on [TIMP-2]*[IGFBP7] in the adult cardiothoracic population was performed to calculate the sample size. We aimed to detect a difference of one unit in [TIMP-2]*[IGFBP7] levels with a standard deviation of 0.8 units [10]. Expected incidence of AKI 2–3 was 10 %. With a given probability of type I error (*α*) of 0.05, and a power (1 − *β*) of 0.9, sample size calculation revealed a required minimum size of 60 patients at total.

### Statistical analysis

Statistical analyses were performed with SPSS Statistics 19 (IBM, Chicago, IL). Continuous data were expressed as mean ± SD; categorical data were expressed as percentage. Comparisons between two groups were carried out using unpaired Student’s *t* test for normally or the Mann–Whitney Rank Sum Test for non-normally distributed data. Multiple groups were compared with ANOVA. Statistical significance was assumed for a *p* value <0.05. To measure the sensitivity and specificity of urinary [TIMP-2]*[IGFBP7] at different cut-off values, a conventional receiver operating characteristic (ROC) curve was generated. The optimal cut-off level was defined by the largest sum of sensitivity and specificity. Comparison of ROC curves was performed with the DeLong-Test.

## Results

### Patients’ characteristics

**Fig. 1:**
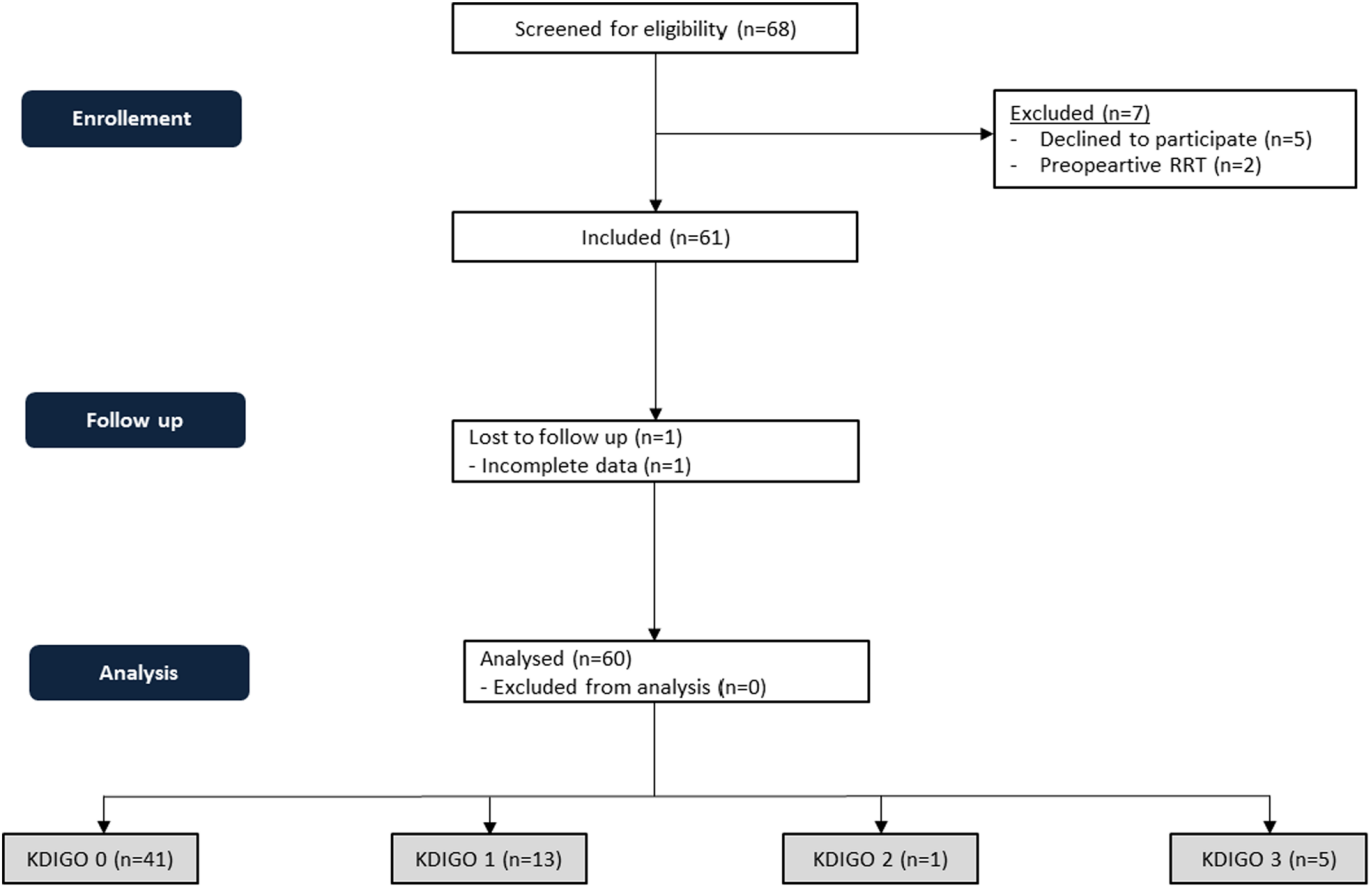
CONSORT (Consolidated Standards of reporting trials) 2010 flow diagram

60 patients undergoing isolated first-time CABG were included in this study (see Fig. 1). AKI of any stage within 48 h after surgery developed in 19 patients (31.7 %): AKI was classified as stage 1 according to KDIGO in 13 patients (21.7 %) and as stage 2 in one patient (1.7 %). Five patients (8.3 %) suffered from AKI stage 3. All of them required renal replacement therapy (RRT) (8.3 %).

The distributions of applied criteria for AKI staging on the basis of serum creatinine or diuresis are given in Table 1. Nine patients were defined as patients with AKI primarily on the basis of increased serum creatinine concentration, and ten patients first reached the threshold of a low diuresis rate.

**Table 1:** Classification criteria for staging of AKI

AKI KDIGO stage	Number	Classification criterion for AKI stage	
		Increase of serum creatinine	Decrease of diuresis or RRT
1	13 (21.7 %)	8	5
2	1 (1.7 %)	1	0
3	5 (8.3 %)	0	5
All (1–3)	19 (31.7 %)	9	10

Pre-procedural characteristics as well as operative variables and postoperative outcomes of patients suffering from AKI 2/3 compared to those without significant postoperative renal dysfunction (KDIGO 2/3) are summarized in Table 2. Patients with renal impairment were significantly older than those without (76.2 ± 3.9 vs. 68.8 ± 9.1, *p* = 0.003). Preoperative renal function assessed by serum creatinine and eGFR showed no difference between the two groups. Other demographics and prevalence of relevant comorbidities were comparable between both groups. Patients with a moderate–severe AKI suffered from a complicated and prolonged postoperative course: compromised hemodynamics corresponding to shock was observed in five patients with AKI 2/3 (83.3 %) and only in six (11.1 %) patients with AKI 0/1 (*p* < 0.001). In addition, incidence of sepsis was higher in patients with moderate to severe AKI [5 (83.3 %) vs. 3 (5.6 %), *p* < 0.001]. Consecutively, among the AKI 2/3 group ICU stay tended to be longer without reaching statistical significance (ICU stay: 9.7 days ± 6.4 vs. 5.5 ± 7.6, *p* = n.s.). Hospital mortality was significantly higher in patients with AKI 2/3 [3 (50.0 % vs. 0 (0 %), *p* < 0.001]. Among the three survivors with AKI2/3, renal function recovered within 6 months after surgery without need for long-term RRT.

**Table 2:** Patient characteristics of patients with AKI KDIGO ≥2 compared to patients with no or mild AKI (KDIGO 0–1)

	AKI 2/3 (*n* = 6)	AKI 0/1 (*n* = 54)	*p* value
Age (years)	76.2 ± 3.9	68.8 ± 9.1	0.003
Male gender (*n*, %)	5 (83.3)	43 (79.6)	n.s.
Weight (kg)	77.8 ± 11.1	83.7 ± 17.7	n.s.
Height (cm)	174.3 ± 9.2	171.8 ± 8.3	n.s.
CPB time (min)	113.3 ± 41.8	102.1 ± 47.3	n.s.
Preoperative Creatinine (mg/dl)	1.01 ± 0.17	1.14 ± 0.23	n.s.
Preoperative eGFR (ml/min/1.73 m^2^)	73.5 ± 13.9	65.0 ± 17.5	n.s.
SAPS	30.8 ± 11.6	24.9 ± 13.8	n.s.
Diuresis on the day of CABG (ml/kgBW/h)	1.30 ± 0.33	0.92 ± 0.69	n.s.
Diuresis on POD 1 (ml/kgBW/h)	1.69 ± 0.66	1.27 ± 0.49	n.s.
Fluid balance on the day of surgery (ml)	2843.0 ± 973.8	2354.2 ± 867.5	n.s.
TISS-28	26.0 ± 6.2	19.7 ± 10.4	n.s.
Comorbidities (*n*, %)			
MI within 6 months	3 (50.0)	13 (24.1)	n.s.
Heart failure NYHA ≥3	1 (16.7)	3 (5.6)	n.s.
Hypertension	4 (66.7)	49 (90.7)	n.s.
Smoking	3 (50.0)	21 (38.9)	n.s.
Diabetes	2 (33.3)	19 (35.2)	n.s.
COPD	1 (16.7)	7 (12.9)	n.s.
Atrial fibrillation	4 (66.7)	21 (38.9)	n.s.
Peripheral artery disease	1 (16.7)	17 (31.5)	n.s.
Shock (*n*, %)	5 (83.3)	6 (11.1)	<0.001
Sepsis (*n*, %)	5 (83.3)	3 (5.6)	<0.001
ICU stay (days)	9.7 ± 6.4	5.5 ± 7.6	n.s.
Hospital mortality (*n*, %)	3 (50.0)	0 (0)	<0.001

*COPD* chronic obstructive pulmonary disease, *SAPS* Simplified Acute Physiology Score, *TISS-28* Therapeutic Intervention Scoring System 28, *POD* postoperative day

### Postoperative course of biomarkers

**Fig. 2:**
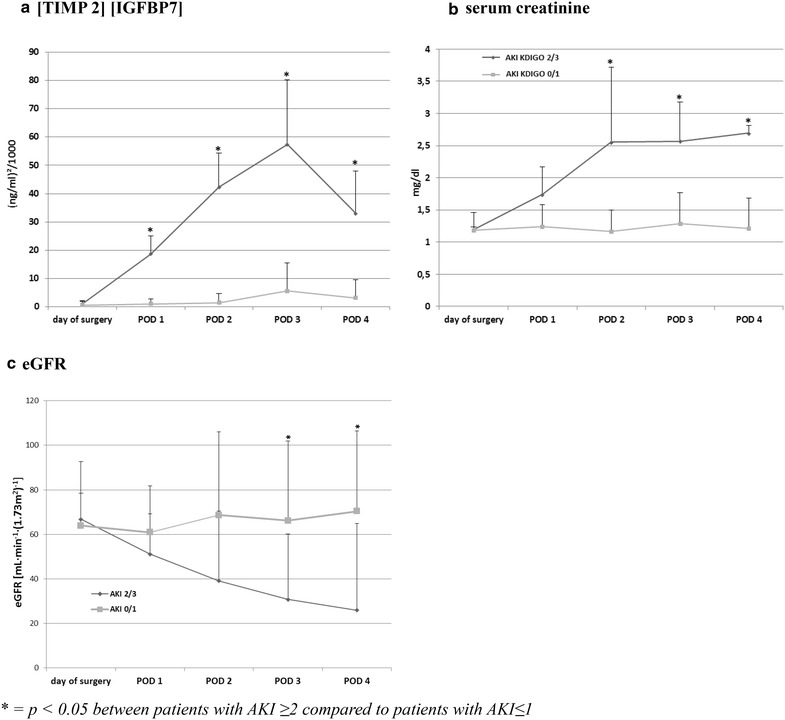
Postoperative course of [TIMP-2]*[IGFBP7] (**a**) serum creatinine (**b**) and (**c**) eGFR in patients with AKI ≥2 compared to patients with AKI ≤1

The postoperative course of serum creatinine, eGFR and urinary [TIMP-2]*[IGFBP7] for patients with AKI stage 2/3 and those with no or mild AKI is illustrated in Fig. 2. No significant rise in urinary [TIMP-2]*[IGFBP7] was observed in patients with AKI 0/1 at any time indicating that surgical myocardial revascularization per se has no influence on the investigated G1 cell cycle arrest biomarkers.

In patients developing AKI 2/3 within the 48 postoperative hours, [TIMP-2]*[IGFBP7] increased significantly already 4 h after surgery compared to patients with AKI 0/1. Notably, a maximum on the 3rd day with an increase to 57.4 ± 32.8 (ng/ml)^2^/1000 could be observed.

In patients with AKI 0/1, serum creatinine as well as eGR remained stable at all times with no significant undulations over time. In contrast, in patients with AKI 2/3, elevated serum creatinine levels could be observed from postoperative day (POD) 2 to POD 4 with a maximum of 2.6 ± 1.1 mg/dl as well as elevated eGFR on POD 3 and 4.

**Fig. 3:**
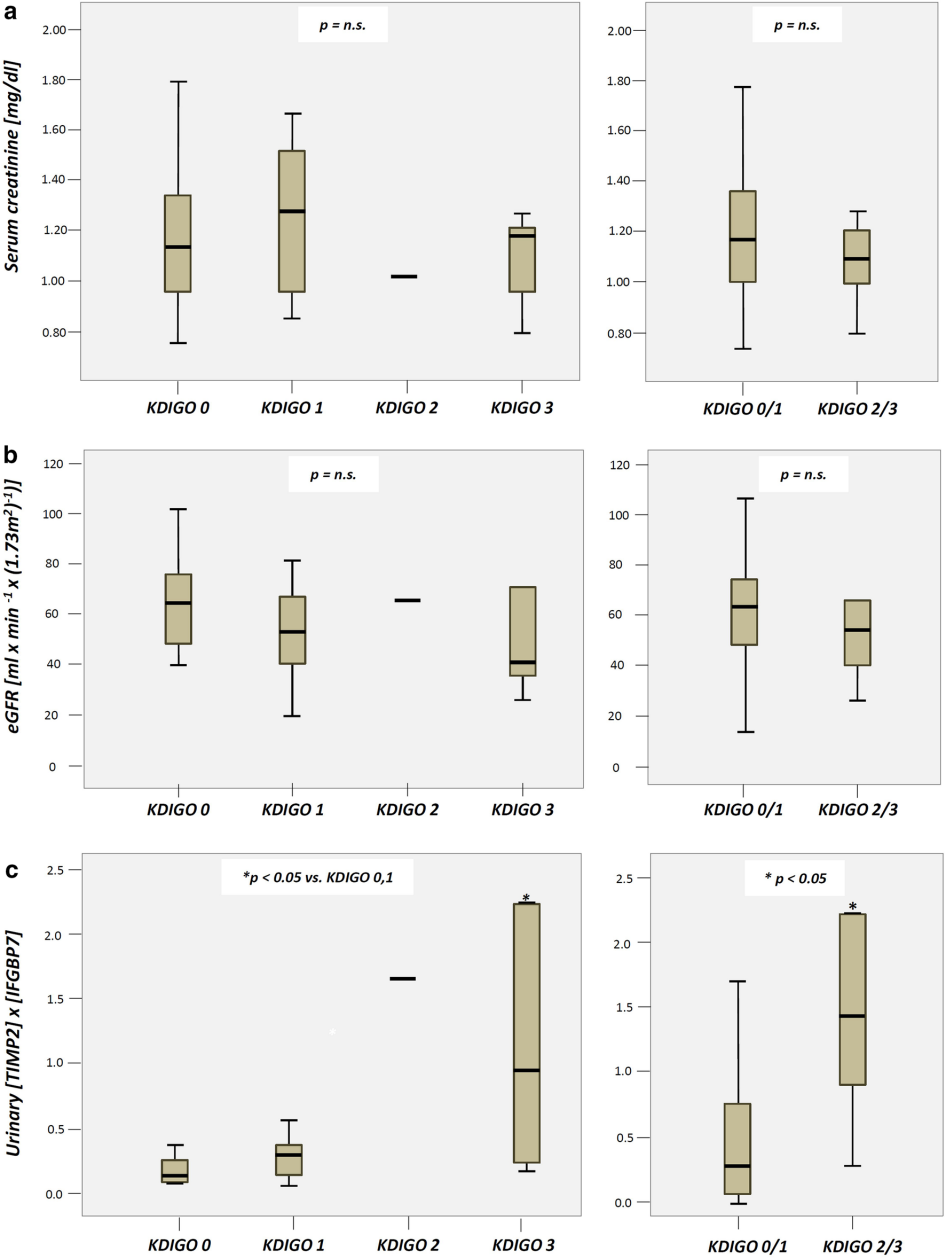
Boxplots of biomarker values on postoperative day 1 after CABG grouped by KDIGO stage. **a** Serum creatinine, **b** eGFR, **c** urinary [TIMP 2]*[IGFBP7]

Distribution of biomarkers measured on POD 1 after surgery according to the KDIGO classification is shown in Fig. 3. Urinary [TIMP-2]*[IGFBP7] values in patients with AKI 3 (median 0.92, range 0.13–5.93) were significantly higher than in patients with AKI 0 (median 0.065, range 2.69) or AKI 1 (median 0.23, range 0.02–0.53). Accordingly, patients with AKI 2 or 3 showed significantly higher values for [TIMP-2]*[IGFBP7] than patients with AKI 0–1 (median 1.29, range 0.13–5.93 vs. 0.10, range 0.02–2.69, *p* < 0.05).

In contrast, serum creatinine as well as eGFR levels did not differ significantly between subgroups stratified by KDIGO.

### Prediction of AKI with biomarkers

**Fig. 4:**
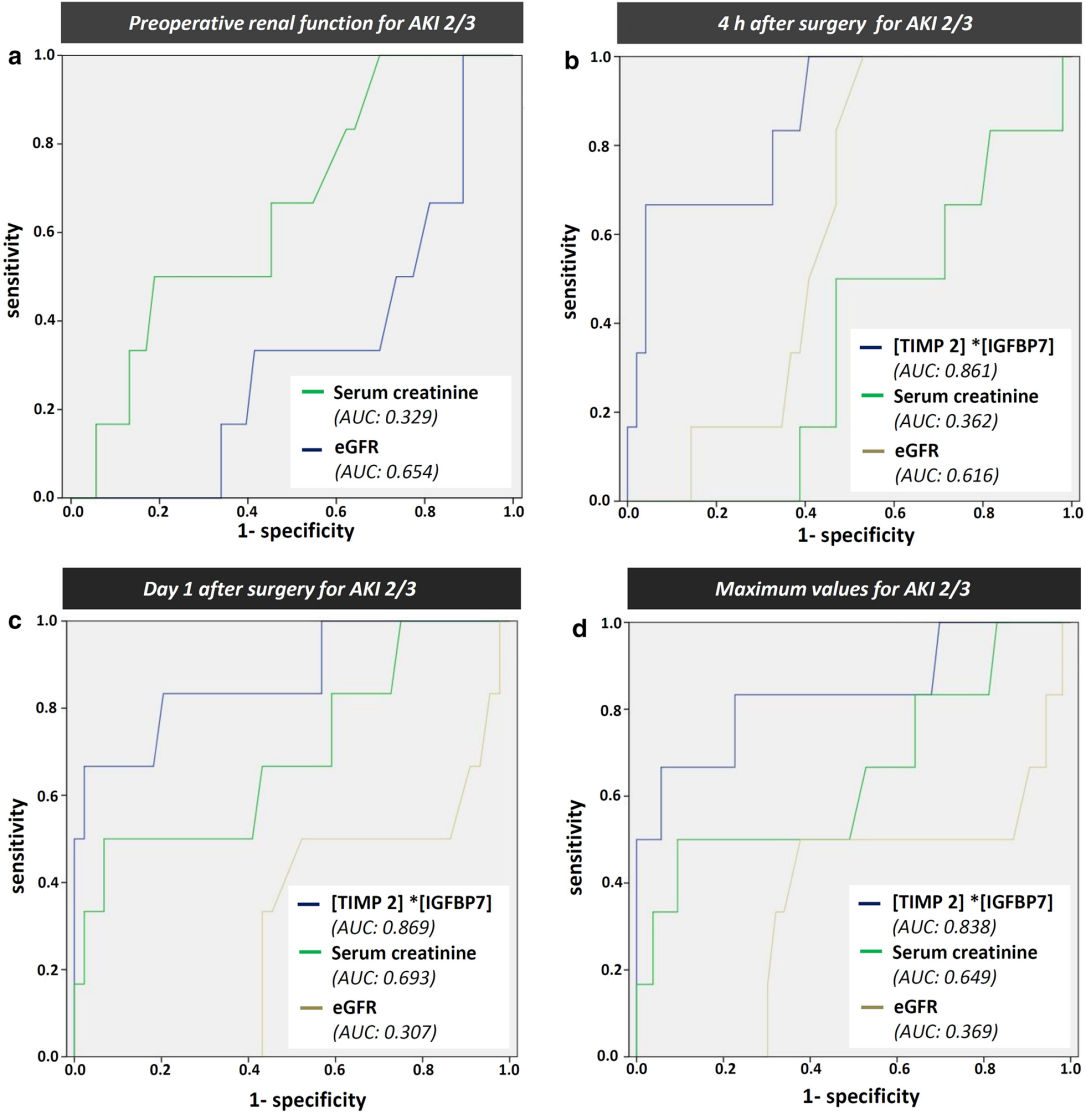
Receiver operator characteristic (ROC) curve for the prediction of AKI 2–3. **a** Preoperative serum creatinine and eGFR, **b** urinary [TIMP-2]*[IGFBP7], eGFR and serum creatinine 4 h after CABG. **c** urinary [TIMP-2]*[IGFBP7], eGFR and serum creatinine on postoperative day 1 after CABG. **d** Maximum urinary [TIMP-2]*[IGFBP7], eGFR and serum creatinine during the first 24 h after CABG

Using ROC analyses, preoperative serum creatinine (AUC 0.329, 95 % CI 0.131–0.527, SE 0.101, *p* = 0.127), preoperative eGFR (AUC 0.654, 95 % CI 0.446–0.862, SE 0.106, *p* = 0.219), as well as serum creatinine measured 4 h after surgery (AUC 0.362, 95 %CI 0.165–0.559, SE 0.100, *p* = 0.274) did show low predictive values for AKI stage 2/3 (see Fig. 4).

eGFR 4 h after CABG showed moderate predictive capacity (AUC 0.616, 95 % CI 0.463–0.768, SE 0.78, *p* = 0.359). In contrast, diagnostic accuracy for urinary [TIMP-2]*[IGFBP7] 4 h after CABG was more reliable with an AUC 0.861 (95 % CI 0.717–1.0, SE 0.073, *p* = 0.04), a sensitivity of 0.83 and a specificity of 0.67 when using a cut-off value of 0.15 (Fig. 4).

On the 1st postoperative day, setting a cut-off value of 1.16 mg/dl for serum creatinine revealed a sensitivity and specificity of 0.67 and 0.57, respectively (AUC 0.693, 95 % CI 0.448–0.939, SE 0.088, *p* = 0.128). eGFR on POD 1 showed inferior predictive properties with an AUC of 0.307 (95 %CI 0.091–0.523, SE 0.110, *p* = 0.128).

In contrast, [TIMP-2]*[IGFBP7] on postoperative day 1 showed a higher sensitivity and specificity (0.80 and 0.81, respectively), using a cut-off value of 0.89 (AUC 0.869, 95 % CI 0.696–1.0 SE 0.099, *p* = 0.04). Urinary [TIMP 2]*[IGFBP7] maximum values within 24 h after surgery were associated with a comparable sensitivity (0.83) and specificity (0.78) (AUC 0.838, 95 % CI 0.632–1.0, SE 0.105, *p* = 0.007, cut-off 0.89) with maximum serum creatinine and lowest eGFR being associated with a lower sensitivity and specificity. Table 3 summarizes the predictive values of postoperative [TIMP 2]*[IGFBP7] concentrations at three cut-offs: In addition to the optimal cut-off level from our ROC analyses defined by the largest sum of sensitivity and specificity, we applied previously published cut-off points from the Sapphire study: one cut-off with the highest sensitivity [0.3 (ng/ml)^2^/1000)] and one with the highest specificity [2.0 (ng/ml)^2^/1000].

**Table 3:** Analysis of applying best and previously published cut-off points of urinary [TIMP–2]*[IGFBP7] for the prediction of AKI KDIGO 2/3

AUC	Cut-off	Sensitivity	Specificity
4 h after surgery			
0.861	0.15	0.83	66.7
	0.3	0.67	0.76
	2.0	0.33	0.98
Day 1 after surgery			
0.817	0.89	0.8	0.81
	0.3	1.0	0.07
	2.0	0.4	0.95
Maximum early value^a^			
0.838	0.89	0.83	0.78
	0.3	0.83	0.35
	2.0	66.7	0.94

*AKI* acute kidney injury, *AUC* area under the curve, *IGFBP7* insulin-like growth factor-binding protein 7, *KDIGO* Kidney Disease: Improving Global Outcomes, *RRT* renal replacement therapy, *TIMP-2* tissue metalloproteinase-2

^a^Defined as maximum level in the first 24 h after surgery

## Discussion

In critically ill patients, cardiac surgery with the use of CPB is the second most common cause of AKI after sepsis, with approximately 1–5 % of patients requiring RRT [16]. AKI in the cardiothoracic population is associated with a significant increase of morbidity and mortality, as well as prolonged length of ICU and hospital stay [1].

In the absence of other appropriate alternatives, the use of serum creatinine as standard diagnostic test for acute kidney injury is commonly accepted among intensivists, despite its known major limitation: Renal function must be impaired more than 50 % before an elevation in serum creatinine can be detected [17]. In addition, serum creatinine does not accurately depict kidney function and rapidly changing glomerular fraction rate (GFR) until a steady state has been reached [17]. Among other shortcomings, serum creatinine levels do not only dependent on excretion through glomerular filtration, but also on production mainly by muscle cells and secretion via tubular cells. Because of this, conditions that affect muscle mass and tubular secretion (such as age, gender, race, and level of GFR) may influence the baseline level and the degree of rise in serum creatinine [17]. Moreover, other specific conditions of cardiac surgery with extracorporeal circulation, e.g., the CPB induced hemodilution with a decrease in serum creatinine concentration cumber the creatinine-based diagnosis of AKI [18]. Experimental studies indicate that specific preventive or therapeutic strategies might be able to reduce AKI-associated morbidity and mortality without showing comparable positive results in the clinical setting [19]. This discrepancy might at least partly be explained by the inability to identify AKI at a very early stage. Although Cystatin C and NGAL seem to be reliable in selected patient cohorts and automated assay methods are commercially available, the value of these two promissing biomarkers in the early diagnosis of AKI remains controversial [20–22].

Taken together, there is no biomarker available for early and reliable detection of AKI, in particular after cardiac surgery, with adequate sensitivity and specificity. Recent studies focused on combinations of biomarkers including urinary insulin-like growth factor-binding protein 7 (IGFBP-7) and tissue inhibitor of metalloproteinases-2 (TIMP-2). This takes into account different pathophysiological mechanisms and, thus, different time courses of biomarker release.

IGFBP7 and TIMP-2 are inducers of the G1 cell cycle arrest found in renal tubular cells being considered as a key mechanism of AKI. They have recently emerged as potential novel markers for risk stratification and early identification of kidney stress. In the multicenter observational Discovery study, a total of 300 potential biomarkers for the detection of AKI were evaluated in a heterogeneous group of 522 patients at high risk of AKI [9]. The results for IGFBP7 and TIMP-2 were better than for any other investigated biomarker (including NGAL, KIM-1, and IL-18) with increased sensitivity and specificity when used as combination compared to its single use (AUC of 0.80 vs. 0.76 and 0.79 alone). Whereas neither TIMP-2 nor IGFBP7 was elevated in patients without AKI being unaffected by important co-morbidities such as chronic kidney disease, diabetes, and sepsis, patients with a [TIMP-2]*[IGFBP7] greater than 0.3 had seven times the risk for AKI.

A follow-up trial—the Opal-trial—using identical enrolment criteria replicated the results of the Discovery study confirming TIMP-2 and IGFBP7 to have better performance than existing markers. Sensitivity and specificity of two cut-off values demonstrated the suitability of these cell cycle arrest markers for clinical application [23].

However, etiologies and pathophysiology of AKI differ substantially between patient populations and, therefore, the performance of every biomarker in different patient cohorts and clinical settings may vary significantly requiring careful and specific evaluation of the marker in different scenarios. This also applies for G1 cell cycle arrest marker: IGFBP7 is superior to TIMP-2 in surgical patients whereas TIMP-2 is better for prediction of septic AKI, demonstrating that these two biomarkers are involved in slightly different pathways. Therefore, it is not clear whether the excellent results of the Discovery, Sapphire and Opal study can be transferred to patients undergoing CABG. Studies investigating the role of [TIMP-2]*[IGFBP7] in the prediction of AKI and defining cut-off values in cardiovascular patient populations are rare and currently restricted to adult and pediatric open cardiac surgery. Meersch et al. investigated the diagnostic properties of these biomarkers for the detection of AKI in 50 patients undergoing CABG who were considered to be at high risk for impaired perioperative renal function assessed by the Cleveland Foundation Score [11]. Whereas diagnosis of AKI based on serum creatinine was delayed, TIMP-2 and IGFBP7 increased as early as 4 hours after surgery with a significant difference between patients with AKI and those without. The maximum urinary [TIMP-2]*[IGFBP7] value within the first 24 h following CABG showed excellent diagnostic accuracy with an area under the receiver operating characteristic curve of 0.84. Sensitivity was 0.92, and specificity was 0.81 for a cutoff value of 0.50. In addition, prediction of renal recovery from AKI was possible with urinary [TIMP-2]*[IGFBP7]. Comparable data were published from the same working group in a pediatric cardio surgical cohort (AUC 0.85, sensitivity 0.83, specificity 0.77 for cut-off 0.70 [(ng/ml)^2^/1000] [24].

The cut-off values defined in our study were slightly different from recently published studies In the Discovery, Sapphire and Opal study, best cut-off values for discrimination of patients with high risk for oncoming AKI was 0.3 (ng/ml)^2^/1000 whereas Meersch set the value of 0.5 (ng/ml)^2^/1000 for the adult and 0.70 (ng/ml)^2^/1000 for the pediatric cardiothoracic population. As mentioned above, etiologies and pathophysiology of AKI differ substantially between patient populations and therefore cut-off values from general surgical, septic, or medical patients might not be applicable to cardiothoracic patients.

Even comparing our results with other studies about the utility of urinary [TIMP–2]*[IGFBP7] for diagnosis of AKI after cardiac surgery may be difficult: Incidence of AKI was 52 % in the study from Meersch vs. 31.7 % in our study. In contrast, five patients suffered from AKI 3 in our series compared to one in the cited trial. The incidence of AKI after cardiac surgery depends on the type of surgery varying significantly between isolated coronary artery bypass grafting, valvular surgery and being the highest for combined CABG+ valvular surgery. Merrsch included all types of cardio surgical procedures not giving any detailed information about performed procedures. In contrast, only isolated CABG procedures were included in our study. In addition, type of CBP and myocardial protection strategy might influence incidence as well as time onset of AKI.

If AKI is recognized early, nephroprotective measures can be considered to reduce exposure to renal insults and potentially avoid the development of higher stage AKI. Although the discussion about preventive or therapeutic interventions in critically ill patients with AKI is controversial, there are some strategies that may be beneficial in the ICU setting: Although there are no specific therapies for AKI, the optimization of fluid balance and hemodynamics as well as a medication review with avoidance of nephrotoxic drugs can reduce the incidence and severity of AKI and improve long-term outcomes [7]. This applies in particular for the subgroup of high risk patients that might be identified better with the cell cycle arrest markers. Accordingly, delayed consultation of a nephrologist is associated with higher mortality and increased dialysis dependence rates in critically ill AKI patients at hospital discharge [25]. In addition, earlier commencement of RRT in critically ill patients with incipient AKI may be beneficial with a reduction of mortality, in particular for patients after cardiac surgery [6, 26]. Preoperative remote ischemic preconditioning significantly reduced the rate of AKI and use of RRT in patients undergoing cardiac surgery in a recently published multicenter randomized trial [27]. Therefore, our standard regime includes a bundle of interventions if [TIMP 2]*[IGFBP7] is higher than the reported cut-off value including consultation of a nephrologist, extensive chart review and stop of all nephrotoxic agents, goal-directed hemodynamic management, delay—if possible—of any exposure to contrast agent, discussion of early RRT and delay of transferring the patient from the ICU to the normal ward. Therefore, urinary [TIMP-2]*[IGFBP7] might help to initiate established preventive and therapeutic interventions and investigate new concepts to prevent AKI.

## Conclusion

Urinary [TIMP-2]*[IGFBP7] may be considered as early predictor of AKI after CABG surgery. It allows the identification of patients at high risk for the development of severe AKI, even with need for RRT, within the postoperative course. Urinary G1 cell cycle arrest biomarkers allow the diagnosis of AKI earlier than creatinine-based definition of AKI.

## Limitations

There are some limitations to our study. Due to the relatively small sample size, the predicted cut-off values for [TIMP-2]*[IGFBP7] in the urine may be different from that obtained in other populations.

In the present study, the measurements of the biomarkers were conducted using the commercially available test kit, which only displayed the product of [TIMP-2] and [IGFBP7]. Therefore, we cannot draw any conclusions about the diagnostic properties of TIMP-2 and IGFBP7 alone in comparison to their multiplication.

In the current study, urinary creatinine was not measured to estimate the true creatinine clearance for these patients. There are three major errors that can limit the accuracy of the creatinine clearance as an estimate of GFR: errors in urine collection; increases in both creatinine secretion and extrarenal creatinine degradation as the GFR falls. Because of these limitations, we did not collect 24 h urine to calculate creatinine clearance but used the Cockcroft–Gault formula to estimate GFR.

## Abbreviations

AKI: acute kidney injury, AUC:area under the curve
BMI: Body Mass Index
CABG: coronary artery bypass graft
CAD: coronary artery disease
CBP: cardiopulmonary bypass
COPD: chronic obstructive pulmonary disease
eGFR: estimated glomerular filtration rate
IGFBP7: insulin-like growth factor binding protein 7
ICU: intensive care unit
KDIGO: Kidney Disease: Improving Global Outcomes
LCOS: low cardiac output syndrome
log ES I: logistic EuroScore I
LOS: length of stay
LVEF: left ventricular ejection fraction
PHT: pulmonary hypertension
ROC: receiver operating characteristic
RRT: renal replacement therapy
SAPS: Simplified Acute Physiology Score
TISS: Therapeutic Intervention Scoring System
TIMP-2: tissue metalloproteinases 2
